# Different Volumetric Measurement Methods for Pituitary Adenomas and Their Crucial Clinical Significance

**DOI:** 10.1038/srep40792

**Published:** 2017-01-18

**Authors:** Chi-Cheng Chuang, Shinn-Yn Lin, Ping-Ching Pai, Jiun-Lin Yan, Cheng-Hong Toh, Shih-Tseng Lee, Kuo-Chen Wei, Zhuo-Hao Liu, Chung-Ming Chen, Yu-Chi Wang, Cheng-Chi Lee

**Affiliations:** 1 Institute of Biomedical Engineering National Taiwan University, Taipei, Taiwan, R.O.C; 2 Department of Neurosurgery, Chang Gung Memorial Hospital at Linkou, Taoyuan, Taiwan, R.O.C; 3 Department of Radiation Oncology, Chang Gung Memorial Hospital at Linkou, Taoyuan, Taiwan, R.O.C; 4 Department of Medical Imaging and Intervention, Chang Gung Memorial Hospital at Linkou, Taoyuan, Taiwan, R.O.C; 5 Department of Neurosurgery, Chang Gung Memorial Hospital at Keelung, Keelung, Taiwan, R.O.C; 6 Chang Gung University, Taoyuan, Taiwan, R.O.C

## Abstract

Confirming the status of residual tumors is crucial. In stationary or spontaneous regression cases, early treatments are inappropriate. The long-used geometric calculation formula is 1/2 (length × width × height). However, it yields only rough estimates and is particularly unreliable for irregularly shaped masses. In our study, we attempted to propose a more accurate method. Between 2004 and 2014, 94 patients with pituitary tumors were enrolled in this retrospective study. All patients underwent transsphenoidal surgery and received magnetic resonance imaging (MRI). The pre- and postoperative volumes calculated using the traditional formula were termed A1 and A2, and those calculated using the proposed method were termed O1 and O2, respectively. Wilcoxon signed rank test revealed no significant difference between the A1 and O1 groups (P = 0.1810) but a significant difference between the A2 and O2 groups (P < 0.0001). Significant differences were present in the extent of resection (P < 0.0001), high-grade cavernous sinus invasion (P = 0.0312), and irregular shape (P = 0.0116). Volume is crucial in evaluating tumor status and determining treatment. Therefore, a more scientific method is especially useful when lesions are irregularly shaped or when treatment is determined exclusively based on the tumor volume.

Pituitary tumors account for 10–20% of all primary intracranial tumors, and functioning pituitary adenomas account for 30% of pituitary adenomas. Functioning tumors usually present with endocrine symptoms, and the first-line treatment for most functioning tumors except prolactinomas is surgical removal, mainly transsphenoidal surgery (TSS)^1^. When the surgery is unsuccessful in controlling hormone secretion and tumor proliferation, medical treatment and/or radiation therapy is necessary^1^,^2^,^3^,^4^,^5^,^6^. Nonfunctioning pituitary adenomas (NFPAs) are the most prevalent pituitary adenomas, accounting for 30–40% of pituitary adenomas. NFPAs are widely considered to cause serious clinical symptoms such as visual impairment and pituitary insufficiency by their mass effect. The gross total resection (GTR) of NFPAs should be attempted in order to relieve the mass effect and decompress the optic apparatus and pituitary gland; generally, the GTR rate for NFPAs is approximately 60–70%^7^,^8^ in low-grade Knosp and noninvasive pituitary adenomas. In invasive adenomas, the GTR rate may be as low as 30–60%^9^,^10^. For reducing residual tumor growth after surgery, postoperative external radiation therapy (EXRT) is often employed, despite the necessity, efficacy, and potential complications of this treatment modality being the subject of considerable debate^11^. Park *et al*. adopted the “wait and see” approach, suggesting that withholding radiotherapy for NFPAs after subtotal resection (STR) avoids exposure to the risks associated with radiation^12^. For functioning^13^,^14^ or nonfunctioning tumors^11^, radiotherapy had been considered to provide long-term control, yet more than 50% of patients in related studies have developed hypopituitarism. Although stereotactic radiosurgery (SRS) is a safer and more precise method than radiotherapy, approximately 18–58% of patients experience newly developed endocrine dysfunction within a 5-year-follow-up^15^. Thus, regardless of the tumor type, preventive radiotherapy remains controversial and its benefits must be weighed against its risks.

In the “wait and see” approach^12^, Park *et al*. also mentioned that tumor recurrence can be detected before they become symptomatic through the close examination of magnetic resonance imaging (MRI); therefore, the precise detection and measurement of tumor recurrence and status are crucial. For a long time, only a one-dimensional measurement or the traditional geometric formula has been used to calculate tumor volume for assessing tumor status. The geometric formula, which is 1/2 (length × width × height) [1/2 (L × W × H)], has been used by most clinicians; however, it is not adequately precise, especially for irregularly shaped tumors such as residual and lobulated pituitary adenomas. Such tumors are difficult to precisely measure, leading to the inaccurate assessment of tumor status. Therefore, in this study, we applied two methods—the traditional geometric formula and a more scientific method—using the OsiriX software (www.osirixviewer.com), and examined the differences between the methods and the importance of the precise measurement of tumor volume. In addition, we calculated the tumor volume through another computerized measurement method—the 3D slicer software (www.slicer.org)—and examined the differences between the results of the OsiriX software.

## Results

### Patient Subgroups

Between 2004 and 2014, 94 patients with pituitary macroadenomas underwent a total of 104 procedures; 10 patients underwent a second operation because of residual tumor progression. As shown in Table 1, we performed GTR in 53 cases (51.0%), and STR in the remaining 51 cases (49.0%). High-grade cavernous sinus (CS) invasion was observed in 73 cases (70.2%), and suprasellar extension was identified in 88 of the cases (84.6%). The preoperative tumor shape was irregular in 22 cases (21.1%), whereas the residual tumor shape was irregular in 89 cases postoperatively (85.6%). Fifty-four cases (51.9%) were diagnosed as apoplexy according to preoperative image, intraoperative surgical, or postoperative pathologic findings.

### Wilcoxon Signed Rank Test

Table 2 presents the A1, O1, S1, A2, O2, and S2 measurement results. The mean preoperative volumes were 10.36 (±10.12) mL, 10.23 (±9.29) mL, and 10.28 (±9.38) mL in the A1, O1, and S1 groups, respectively. The mean postoperative volumes were 2.21 (±3.08) mL, 1.6988 (±2.644) mL, and 1.6989 (±2.636) mL in the A2, O2, and S2 groups, respectively. Wilcoxon signed rank testing revealed no significant difference between the A1 and O1 groups (P = 0.1810) but a highly significant difference between the A2 and O2 groups (P < 0.0001). In addition, Wilcoxon signed rank testing revealed no significant differences between the O1 and S1 groups (P = 0.4964) or the O2 and S2 groups (P = 0.4062). Furthermore, the test did not reveal significant differences between the O_12_ and S_12_ groups (P = 0.5560).

### Univariate Logistic Regression and Categorical Analyses

As shown in Table 3, the preoperative volume difference (≥1.54 mL, 1 SE) was associated with significant differences in high-grade CS invasion (P = 0.0312) and preoperative tumor shape (P < 0.0001). No significant difference was related to suprasellar extension (P = 0.2074) or apoplexy (P = 0.1442). As shown in Table 4, chi-square and Fisher’s exact tests showed that regarding the postoperative volume difference (≥1.40 mL, 1 SE), significances were present for the preoperative volume difference (P < 0.0001), extent of resection (P < 0.0001), high-grade CS invasion (P = 0.0312), and preoperative tumor shape (P = 0.0116). The postoperative volume difference exhibited no significant difference in suprasellar extension (P = 0.2977), postoperative tumor shape (P = 0.7300), or apoplexy (P = 0.1928). In addition, as shown in Table 5, in cases with a postoperative volume difference ≥1 SE and preoperative difference <1 SE, we found that the postoperative volume difference was significantly related to the extent of resection (P = 0.0061) but not to high-grade CS invasion (P = 0.3353), suprasellar extension (P = 0.6860), preoperative shape (P = 0.9999), postoperative shape (P = 0.6865), or apoplexy (P = 0.8821). By contrast, as shown in Table 6, in cases with a postoperative volume difference ≥1 SE and preoperative difference ≥1 SE, we found that the postoperative volume difference was significantly related to the extent of resection (P = 0.0054) and preoperative shape (P < 0.0001) but not to high-grade CS invasion (P = 0.1004), suprasellar extension (P = 0.5918), postoperative shape (P = 0.9999), or apoplexy (P = 0.1142).

### Multivariate Logistic Regression Analysis

As shown in Table 7, when considering multiple variables and a postoperative volume difference ≥1 SE, we found a significant difference in the extent of resection (P = 0.0071). No significant difference was present in the preoperative volume difference (P = 0.3160), high-grade CS invasion (P = 0.3777), suprasellar extension (P = 0.9559), apoplexy (P = 0.3089), or preoperative tumor shape (P = 0.2831).

## Discussion

The GTR rate in most pituitary tumors is approximately 60–70%, even with the assistance of intraoperative computed tomography and navigation. Radiation therapy or medication is administered to treat residual tumors after decompression is achieved intraoperatively. Therefore, the detection and management of residual tumors are crucial. However, some residual tumors shrink after a long period, and determining actual tumor growth by using a more precise method would avoid the adverse side effects and waste of medical resources resulting from unnecessary treatment. The “wait and see” policy is also based on the detection of tumor status by closely examining follow-up postoperative MRI. Nevertheless, these traditional methods may underestimate tumor status, thus delaying treatment or overestimating the status; therefore, treatment methods that are more advanced with unnecessary complications are applied, particularly for STR cases in which the residual tumor is typically irregularly shaped, rendering the accurate measurement of tumor status and calculation of tumor volume markedly more challenging. The importance of more precise and scientific measurement of the tumor volume cannot be overemphasized.

### Measurement of Tumor Recurrent or Residual Tumor Growth and The Timing and Indications for Radiation Therapy

As mentioned, patients with NFPAs typically harbor a larger tumor volume, which is correlated with a higher chance of postoperative residual tumors in STR cases^16^,^17^,^18^,^19^ and a higher recurrence rate. Some studies have determined regrowth or recurrence according to several criteria such as a 20% increase in residual tumor volume^20^, a 2-mm increase in one axis^21^,^22^, the enlargement of tumor remnants^23^ compared with the previous imaging study, or any radiographic evidence of tumor recurrent or progression^12^,^24^,^25^,^26^. However, the involved criteria exhibited considerable biases and lacked objectivity because of differing interpretation among radiologists and neurosurgeons and the imprecise measurement of tumor status. Most residual tumors are irregularly shaped, rendering measuring them and defining their status difficult; thus, we should pay more attention to these residual tumors, particularly because such tumors are at a greater risk of regrowth. In addition, according to previous reports, the regrowth rate of these tumors is slow but highly variable [tumor volume doubling time (TVDT) ranges from 1106 to 2566 days]^27^. The enormous variability in TVDT may result from the slow growth rate of recurrent and residual tumors, individual interpretation bias, unscientific one-dimensional measurement, and imprecise calculation of tumor volume by using the traditional geometric formula. Some residual tumors may remain unchanged in size^27^,^28^ whereas others shrink; one study reported that 29% of tumors decreased in size during a long follow-up period without any postoperative radiotherapy^29^. Therefore, the objective and accurate measurement of tumor recurrence and regrowth is crucial for defining tumor status and determining the following treatment strategy, and may change a lot in our clinical judgement and management.

Studies have proved that postoperative radiotherapy provides excellent tumor control^15^,^20^,^21^,^30^,^31^ and hormone-level normalization^32^,^33^,^34^,^35^,^36^,^37^,^38^. However, some studies^12^,^24^,^39^,^40^ have advised against prophylactic radiotherapy in favor of long-term follow-up in order to avoid the side effects of radiotherapy. In addition, radiotherapy or radiosurgery causes hypopituitarism, doing so in approximately 30–60% of cases^11^,^13^,^14^,^24^, as well as optic apparatus damage^13^,^31^,^41^,^42^, cognitive function changes^43^, and secondary malignancies^11^,^25^,^40^,^41^,^42^,^44^,^45^. No straightforward benefit of postoperative radiotherapy is apparent for individual patient care; therefore, radiation therapy should be applied until tumor growth is definitely demonstrated. Precisely measuring the tumor volume and defining the status are crucial.

### Preoperative Volume Differences

In our study, Wilcoxon signed rank testing did not reveal a significant difference between the O1 and A1 groups (P = 0.1810). Most of the preoperative tumors, although large in size, were regularly shaped. In such regular-shaped masses, the exact tumor volume could be approximately calculated by 1/2 (L × W × H). The traditional geometrical method was a long-used method for the calculation of an ellipsoidal lesion. The lack of significance in the Wilcoxon signed rank test indicated no difference between the results obtained using the traditional geometric and OsiriX methods and that we could rely on the OsiriX software in counting the volume of the given masses. For ellipsoidal lesions, we expected that the geometrical method and segmentation process would generate similar results because of the regular shape of the tumor and because the OsiriX method can calculate tumor volume even more accurately on the basis of this result. Furthermore, the statistical results shown in Table 3 suggest that the OsiriX method is more accurate than the traditional method in calculating preoperative tumor volumes is.

**Figure 1:**
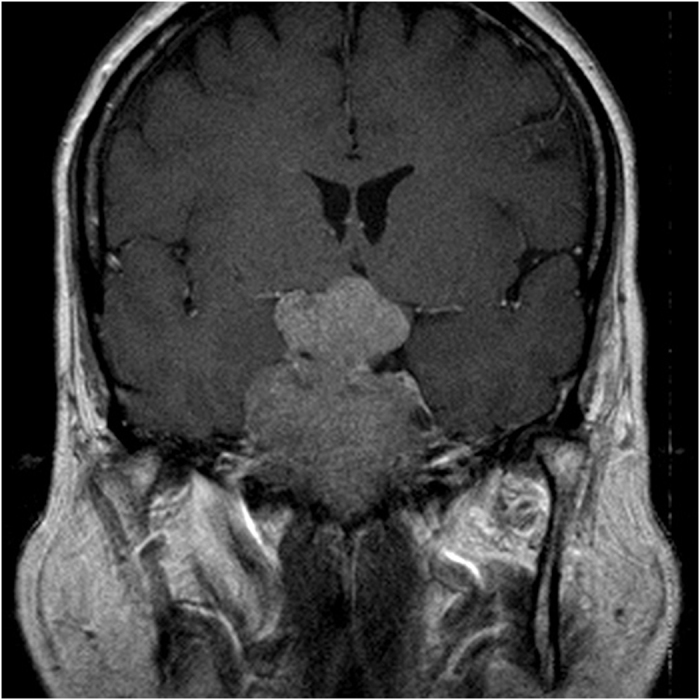
Preoperative MRI of a 64-year-old female patient revealed an extremely large tumor.

**Figure 2:**
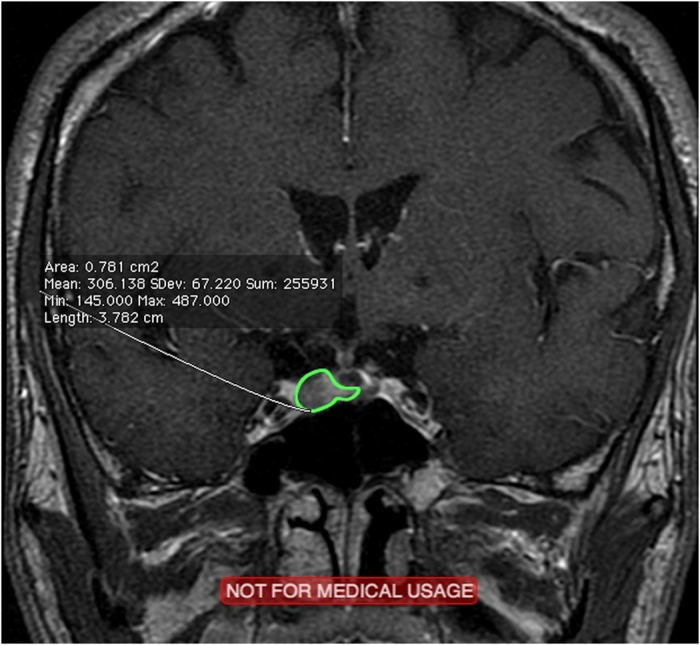
Preoperative MRI of a 57-year-old male patient revealed an extremely small tumor (green area).

**Figure 3:**
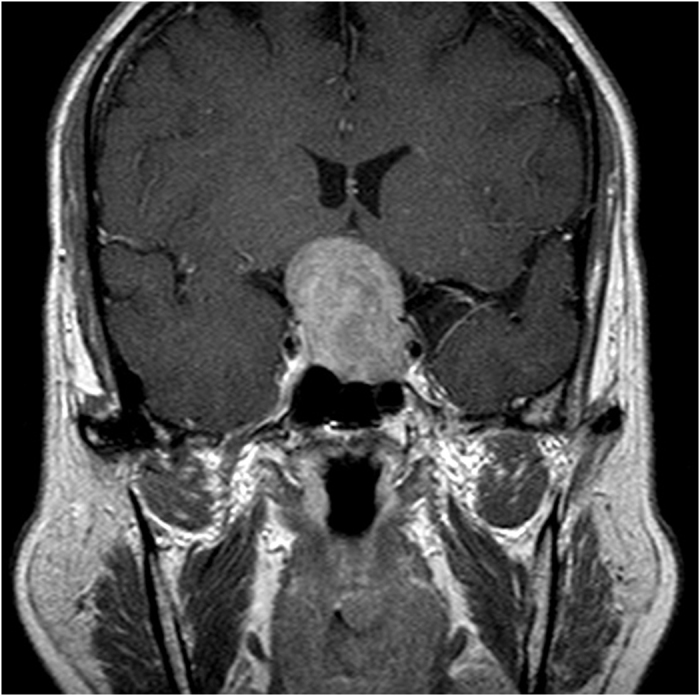
Preoperative MRI of a 62-year-old female patient revealed a typical NFPA with a regularly shaped tumor.

Eleven unusual cases were identified in this study, as shown in Table 3. In these cases, in which the preoperative volume difference ≥1 SE, we noted significant differences in high-grade CS invasion (P = 0.0312) and preoperative tumor shape (P < 0.0001) but not in suprasellar extension (P = 0.2074) and apoplexy (P = 0.1442). Therefore, as mentioned, although there was no significance existed between the O1 and A1 groups, we should apply the more accurate proposed method for patients who have tumors with high-grade CS invasion and/or irregular lobulated shapes, which in this study were most encountered in those with extremely large (Fig. 1) or small preoperative tumors (Fig. 2). In this scenario, we may expect a significant difference in such cases if we calculate the volume by using the two methods, despite no significance between the O1 and A1 groups. As a result, in regular-shaped cases, the traditional geometrical method could be used; however, in invasive or irregular lobulated cases, a more accurate method should be used instead. Regarding suprasellar extension, we believe that the tumors extending suprasellarly, although larger in size, exhibited a more regular shape (Fig. 3) and thus were not associated with a significant preoperative volume difference (≥1 SE). Furthermore, no significant difference was present in apoplexy. We propose two reasons for this result. First, the definition of apoplexy was broad (preoperative images and intraoperative surgical and postoperative pathologic findings) and the degrees of apoplexy were diverse and subjective, rendering the diagnosis nonspecific. Second, the tumors with apoplexy tended to be regular-shaped, and even in cases with high-grade CS invasion or irregular shape, apoplexy seemed to be only the result caused by these preceding predominant factors. Therefore, the effect of apoplexy on postoperative volume differences was not markedly significant.

### Postoperative Volume Differences

The Wilcoxon signed rank testing revealed significant postoperative differences (P < 0.0001) between the A2 and O2 groups. These results suggested that tumor volume varies greatly depending on measurement method, particularly for postoperative volume, and thus should be evaluated using only the most accurate method. As shown in Table 4, in the cases with a postoperative volume difference ≥1 SE, we found significant differences among the preoperative volume difference (P < 0.0001), extent of resection (P < 0.0001), high-grade CS invasion (P = 0.0312), and preoperative tumor shape (P = 0.0116). No significant differences in the postoperative volume difference regarding suprasellar extension (P = 0.2977), postoperative tumor shape (P = 0.7300), and apoplexy (P = 0.1928). We could predict additional significant errors if we used only the 1/2 (L × W × H) formula for measuring tumors with high-grade CS invasion and/or irregular shapes, which were the most common tumors among both the extremely large and small preoperative tumors, especially in those with previous preoperative tumor volume differences. In smaller and less regular-shaped tumors, the volume was difficult to measure accurately by exclusively using the 1/2 (L × W × H) formula. Furthermore, as mentioned, the smaller and less regular-shaped a tumor is, the greater the disparity in tumor volume calculation is, especially when the traditional method or individual measurement criteria are used.

**Figure 4:**
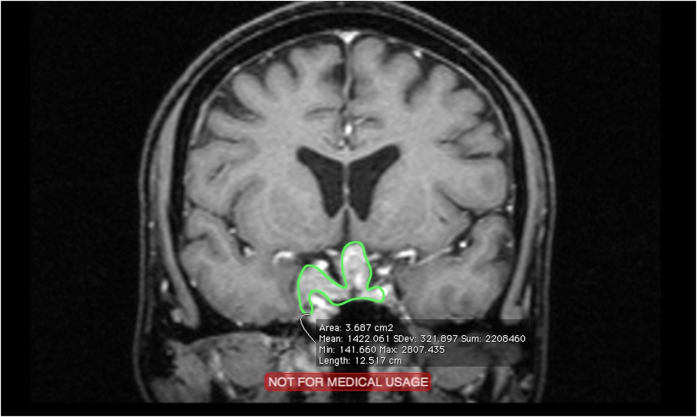
Postoperative MRI of a 55-year-old male patient revealed an irregularly shaped residual tumor with CS invasion after STR (green area).

**Figure 5:**
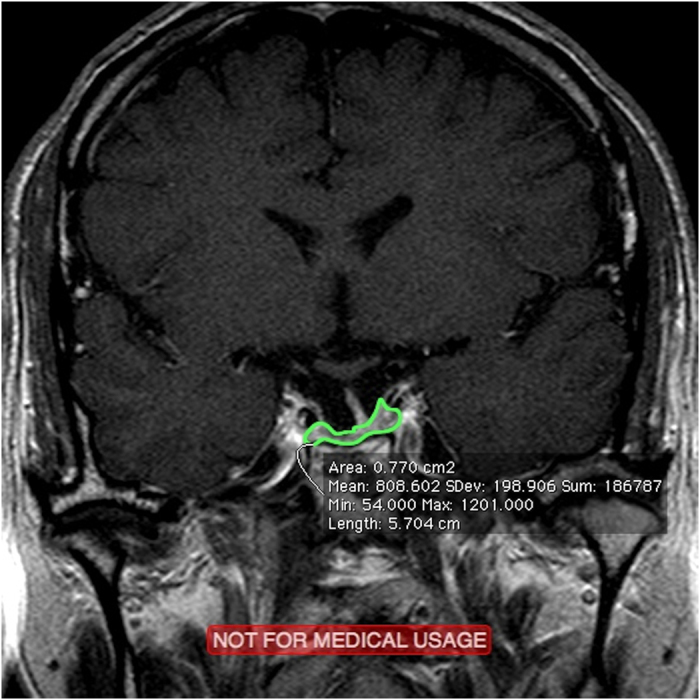
GTR status was achieved in a 60-year-old male patient, the postoperative MRI of whom revealed no tumor remnants but did exhibit a regularly shaped pituitary gland and granulation tissue (excluding the stalk; green area).

Regarding the extent of resection, the STR cases, particularly those involving larger tumors with high-grade CS invasion (Fig. 4), clearly exhibited more residual irregular-shaped remnants, which render tumor volume calculation more difficult and imprecise. Therefore, in larger and more irregularly shaped tumors in which only STR is achieved, a more accurate method should be adopted for measuring the residual tumor status in order to determine a more appropriate treatment strategy. Suprasellar extension was not associated with a significant postoperative volume difference (≥1 SE), because of the aforementioned relatively regular shape. The statistical result between postoperative tumor shape (P = 0.7300) and postoperative volume difference was not as significant as preoperative shape (P = 0.0116) was, possibly because in the GTR cases, we exclusively measured the volume of the gland itself and the granulation tissue, and that in these cases, although the volume did not include the stalk, we still defined the postoperative shape as irregular (Fig. 5). Therefore, the definition of postoperative tumor regularity was ambiguous, and the difference between postoperative shapes was not significant. However, as mentioned, the more irregularly shaped the tumor was, the greater the bias would be if we calculated volume by using only traditional methods. Regarding apoplexy, we propose two reasons for this nonsignificant result. First, as mentioned, the definition of apoplexy was not limited and specific. Second, we believe that in such cases, the tumor may easily shrink once we started to decompress and drain the intratumoral hemorrhage. Therefore, even in the STR cases, the amount of residual tumor, particularly in the CS portion, was low, and the shape of the postoperative residual tumor could be regular or irregular, depending on the individual situation. Therefore, we could expect no significance regarding apoplexy.

### Postoperative Volume Differences in Distinct Groups

As shown in Table 5, cases with a postoperative volume difference ≥1 SE and preoperative difference <1 SE were significantly related to only the extent of resection (P = 0.0061), exhibiting no significant differences in high-grade CS invasion (P = 0.3353), suprasellar extension (P = 0.6860), preoperative shape (P = 0.9999), postoperative shape (P = 0.6865), or apoplexy (P = 0.8821). Thus, in the tumors with a preoperative difference <1 SE, after adjusting for preoperative volume difference, the significant differences in Table 4 (high-grade CS invasion, P = 0.0312; preoperative shape, P = 0.0116) would clearly no longer exist. However, the extent of resection remained a significant factor associated with a postoperative volume difference. Similarly, as stated, this result could be because the STR cases comprised more residual irregularly shaped remnants, which render tumor volume calculation more difficult and imprecise when the traditional method is used. As shown in Table 6, in cases with a postoperative volume difference ≥1 SE and preoperative difference ≥1 SE, we found a significant association with the extent of resection (P = 0.0054) and preoperative shape (P < 0.0001) but not with high-grade CS invasion (P = 0.1004), suprasellar extension (P = 0.5918), postoperative shape (P = 0.9999), or apoplexy (P = 0.1142). As mentioned, in STR and preoperative irregularly shaped cases with previous preoperative volume differences, the tumor remnants are so irregularly shaped that a more precise method should be used to measure them. In summary, regardless of other characteristics, the extent of resection was the most crucial factor influencing the differences between the two calculation methods. In addition, as shown in Table 7, when considering multiple variables and a postoperative volume difference ≥1 SE, we found a significant difference only in the extent of resection (P = 0.0071). This result reinforced the importance of the extent of resection, especially in STR cases associated with irregularly shaped pre- and postoperative tumors as well as high-grade CS invasion. Furthermore, in such STR cases, some patients may require radiation therapy if the remnants grow; therefore, we should closely monitor STR cases and use methods that are more accurate to evaluate tumor volume and status.

Consequently, in preoperative tumors with regularly shaped tumors, we could use the 1/2 (L × W × H) formula; however, tumors with high-grade CS invasion and an irregular shape should not be measured using only the simple traditional method. Most crucially, most residual tumors are irregularly shaped postoperatively and the following treatment is exclusively determined according to the measurement of tumor volume and assessment of tumor status; therefore, we suggest that all tumors, both in STR and GTR cases, should be calculated using the precise method, which will yield the most tailored personal therapeutic options.

### Volume Estimation Results of the OsiriX and 3D Slicer Methods

The OsiriX^46^,^47^ and 3D slicer^48^,^49^ methods have been used to estimate the tumor volume. Other software also demonstrated high reliability^50^,^51^,^52^,^53^,^54^. Riley GT *et al*.^55^ compared the slicer software with the ellipsoid-based method in diffuse pediatric pontine glioma and suggested that the volume calculated slice-by-slice using the slicer software was more suitable for complex-shaped tumors. In our department, we have employed the OsiriX software for a long time and we believe in its accuracy^56^. Therefore, in addition to comparing the OsiriX software with the traditional method, we compared and discussed the performance of the OsiriX and slicer methods in estimating the tumor volume, especially for irregular-shaped tumors, which are mostly encountered in postoperative scenarios.

As shown in Table 2, the Wilcoxon signed rank testing did not reveal a significant difference between the O1 and S1 groups (P = 0.4964), indicating that these methods produced no differences in tumor-volume measurement in preoperative cases, which are often regular-shaped, well-defined, and homogenous. Moreover, the 3D slicer GrowCut segmentation method was substantially more convenient. Most importantly, the Wilcoxon signed rank testing revealed no significant difference between the O2 and S2 groups (P = 0.4062), suggesting that in postoperative scenarios these methods measured irregular-shaped tumors with equal accuracy. Furthermore, because the values of O_12_ and S_12_ represented the tumor volume between the pre- and postoperative conditions for the tested methods, they also represented the volume of the intraoperatively resected tumor, which should be identical in clinical scenarios regardless of the choice of software. The Wilcoxon signed rank testing revealed no significant differences between the O_12_ and S_12_ groups, which confirmed the similarity of volumes obtained through the OsiriX and 3D slicer methods.

Although the GrowCut method is faster and more convenient, invasive, lobular, and irregular-shaped tumors, especially in postoperative cases, should be inspected in detail. Therefore, we suggest evaluating and measuring these tumors slice-by-slice, particularly when the future treatment is determined on the basis of the tumor status.

### Study Limitations

We believe that potentially significant factors associated with postoperative irregular shape are not restricted to surgery. Therefore, our definition of tumor shape seemed to be subjective and vague. In addition, the definition of apoplexy in our study was broad and unclear. Consequently, shape and apoplexy should be more objectively and precisely defined in the future study.

## Materials and Methods

### Patient Population

Ninety-four patients with pituitary macroadenomas who underwent a total of 104 operations between 2004 and 2014 were enrolled in this study. Ten of them underwent a second operation because of residual tumor progression. All the patients underwent 3-mm thin-cut MRI, and routine postoperative MRI was performed within 3 months after surgery and annually thereafter. We calculated the preoperative original and postoperative residual tumor volume by using two methods according to the MRI, and compared the results for each patient. We used the traditional geometric method with the formula 1/2 (L × W × H) to calculate the tumor volume. In addition, we attempted to develop a more scientific method by using the software OsiriX to measure the volume. Signed informed consent was provided by each patient and approval for this study was obtained from the Institutional Review Board of Chang Gung Memorial Hospital, and all methods were performed in accordance with the relevant guidelines and regulations.

### Transsphenoidal Surgery

The surgery was routinely performed under general anesthesia and accompanied by pure endonasal endoscopic TSS by using 0° and 30° 4-mm rigid endoscopes (Karl Storz, Tuttlingen, Germany), which displayed the surgical site on a monitor.

### Treatment Criteria for Recurrent and Residual Tumors

If a residual tumor was detected immediately after TSS or if a recurrent tumor was close to the optic apparatus and produced mass effect, we provided EXRT or SRS immediately after the operation to control the progression. However, if the distance from a residual or recurrent tumor to the optic apparatus was ≥2 mm, we delayed possible radiation side effects by not conducting EXRT or SRS until apparent tumor progress was demonstrated in serial follow-up radiologic images.

### Radiologic Follow-Up

All the patients received pre- and postoperative MRI. After pituitary tumor resection, the packing materials, postoperative debris, thickened mucosa, and blood can interfere with imaging interpretation; however, these postoperative changes are resolved within 3–4 months after surgery. Thus, it is recommended that the effectiveness of surgery be assessed approximately 3 months after initial surgery and annually thereafter for long-term follow-up. Subsequent surveillance imaging studies were conducted at 1-year intervals for 2–3 years and then at increasing intervals. However, patients with residual tumors received more frequent follow-up examinations.

### Imaging Interpretation

A surgeon interpreted the pre- and postoperative MRI. Subsequently, a neuroradiologist, who was blinded to the earlier reading, provided an independent retrospective evaluation of the MRI to reduce reporting bias.

### Subgroups and Tumor Volume Calculation

Ninety-four patients received 104 operations, and we calculated four tumor volumes for every operation. The pre- and postoperative volumes calculated using the traditional geometric formula 1/2 (L × W × H) were termed A1 and A2, respectively, those calculated using the OsiriX software method were termed O1 and O2, respectively, and those calculated using the 3D slicer software method were termed S1 and S2, respectively. The differences between the O1 and A1, O2 and A2, O1 and S1, and O2 and S2 groups were termed Diff (O1 − A1), Diff (O2 − A2), Diff (O1 − S1), and Diff (O2 − S2), respectively. In addition, O_12_ represented the volume difference of the pre- and postoperative status calculated through the OsiriX method (i.e., intraoperatively resected tumor volume), and S_12_ represented the volume differences of the pre- and postoperative status calculated by the 3D slicer method. Furthermore, the value difference between the O_12_ and S_12_ groups was termed Diff (O_12_ − S_12_). When using the OsiriX method, we brushed the tumor area slice-by slice, and at the end of the segmentation process, all the regions of interest (ROIs) were grouped and the volume was computed. When using the 3D slicer method, we applied the GrowCut method to more regular-shaped, well-defined, and homogenous tumors. For less regular-shaped tumors, particularly in postoperative cases, we also brushed the tumor area slice-by-slice. Similarly, at the end of the segmentation process, all ROIs were grouped and the volume was computed. To reduce the measurement bias, the final volume was obtained from the average of the tumor volume calculated from axial, sagittal, and coronal images. When using the traditional formula to calculate postoperative residual tumors, most of which were irregularly shaped, we used the longest diameter for each dimension, as we did in calculating regularly shaped masses. “Regular” was defined as round or ellipsoidal-shaped, and “irregular” was defined as lobulated or unevenly shaped with at least one projection. The presence of a Knosp classification grade III or IV adenoma was defined as indicating high-grade CS invasion. In GTR cases in which the postoperative MRI exhibited only pituitary gland and granulation tissue without any residual tumor remnants, we measured exclusively the volume of the gland itself and the granulation tissue, excluding the pituitary stalk. The significant volume difference was defined as ≥1 standard error (SE); that is, ≥1.54 mL in preoperative cases and ≥1.40 mL in postoperative cases. We chose 1 SE because in different groups the individual cut-off value should represent the volume difference in each group rather than the consistent value.

### Statistical Analysis

The assumption of normal distribution of the calculated tumor volume value was rejected. Therefore, the Wilcoxon signed rank test was used to determine the difference between the A1 and O1, A2 and O2, O1 and S1, and O2 and S2 groups instead of paired *t*-tests. The chi-square test and Fisher’s exact test for independence were used to determine the significance of the differences in the calculated tumor volumes between different categorical variables. Multiple logistic regression analysis was performed to determine which variables were significantly associated with the differences in the calculated tumor volumes. In all cases, a difference was considered significant if P < 0.05. All analyses were conducted using SAS (Statistical Analysis System, version 9.3; SAS Institute Inc., Cary, NC, USA).

## Conclusion

Tumor volume plays a crucial role in determining the initial treatment, tumor status, and subsequent management. The traditional formula for calculating tumor volume is not sufficiently precise, and its use may result in residual tumor growth being overlooked or overestimated. Therefore, we suggest using a more scientific and precise method, especially in STR cases or when the lesions are irregularly shaped or high-grade CS invasion is present. Appropriate and timely treatment should be administered until tumor regrowth is definitively demonstrated in order to avoid unnecessary complications.

## Additional Information

**How to cite this article**: Chuang, C.-C. *et al*. Different Volumetric Measurement Methods for Pituitary Adenomas and Their Crucial Clinical Significance. *Sci. Rep.* **7**, 40792; doi: 10.1038/srep40792 (2017).

**Publisher's note:** Springer Nature remains neutral with regard to jurisdictional claims in published maps and institutional affiliations.

## Figures and Tables

**Table 1:** Baseline characteristics, number of procedures = 104.

Characteristics	N (%)
Extent of resection	
Gross total	53(51.0)
Subtotal	51(49.0)
High grade cavernous sinus invasion	
No	31(29.8)
Yes	73(70.2)
Suprasellar extension	
No	16(15.4)
Yes	88(84.6)
Preoperative shape	
Irregular	22(21.1)
Regular	82(78.9)
Postoperative shape	
Irregular	89(85.6)
Regular	15(14.4)
Apoplexy	
No	50(48.1)
Yes	54(51.9)

**Table 2:** Volume estimation and the results of Wilcoxon signed rank test.

Volume estimate	Mean(SD), mL	Median(range), mL	Interquartile range(Q1–Q3), mL	Normal distribution assumption	Wilcoxon signed rank test for H: diff = 0, p value
O1	10.24(9.29)	8.19(0.82–56.35)	7.26(4.52–11.78)	Rejected	n/a
O2	1.6988(2.644)	0.40(0.22–12.18)	1.07(0.35–1.42)	Rejected	n/a
A1	10.36(10.12)	7.80(0.68–67.37)	7.74(4.51–12.25)	Rejected	n/a
A2	2.21(3.08)	0.88(0.17–20.57)	1.75(0.56–2.31)	Rejected	n/a
S1	10.28(9.38)	8.37(0.88–57.15)	7.29(4.67–11.96)	Rejected	n/a
S2	1.6989(2.636)	0.41(0.22–12.06)	1.06(0.36–1.42)	Rejected	n/a
Diff (O1 − A1)	−0.12(1.54)	0.05[(−11.02)−2.22]	0.64[(−0.22)−0.42]	Rejected	0.1810
Diff (O2 − A2)	−0.51(1.40)	−0.32[(−10.34)−2.52]	0.71[(−0.69)−0.02]	Rejected	<0.0001
Diff (O1 − S1)	−0.04(0.29)	−0.005[(−1.36)−0.61]	0.18[(−0.10)−0.08]	Rejected	0.4964
Diff (O2 − S2)	−0.0002(0.0456)	0[(−0.20)−0.21]	0.02[(−0.01)−0.01]	Rejected	0.4062
Diff (O_12_ − S_12_)	−0.43(2.9985)	−0.10[(−1.38)–0.59]	0.2[(−0.11)−0.09]	Rejected	0.5560

^*^Diff: Difference.

**Table 3:** Univariate analysis of categorical variables using chi-square and Fisher’s exact tests: association between factors and preoperative volume differences (O1 − A1) ≥1SE (1.54 mL).

Characteristics	Preoperative volume difference (O1 − A1) ≥1 SE, n (%)		
	< 1.54 mL	≥1.54 mL	P value
High grade cavernous sinus invasion			0.0312*
No	31(100.0)	0(0.0)	
Yes	62(84.9)	11(15.1)	
Suprasellar extension			0.2074*
No	16(100.0)	0(0.0)	
Yes	77(87.5)	11(12.5)	
Preoperative shape			<0.0001*
Irregular	14(63.6)	8(36.4)	
Regular	79(96.3)	3(3.7)	
Apoplexy			0.1442
No	47(94.0)	3(6.0)	
Yes	46(85.2)	8(14.8)	

^*^Fisher’s exact test.

**Table 4:** Univariate analysis of categorical variables using chi-square and Fisher’s exact tests: association between factors and postoperative volume differences (O2 – A2) ≥1SE (1.40 mL).

Characteristics	Postoperative volume difference (O2 − A2) ≥1 SE, n (%)		
	< 1.40 mL	≥1.40 mL	P value
Preoperative volume difference (O1 − A1) ≥1 SE (1.54 ml)			<0.0001*
<1 SE	80(86.0)	13(14.0)	
≥1 SE	4(36.4)	7(63.6)	
Extent of resection			<0.0001
Gross total	51(96.2)	2(3.8)	
Subtotal	33(64.7)	18(35.3)	
High grade cavernous sinus invasion			0.0312
No	29(93.6)	2(6.4)	
Yes	55(75.3)	18(24.7)	
Suprasellar extension			0.2977*
No	15(93.8)	1(6.2)	
Yes	69(78.4)	19(21.6)	
Preoperative shape			0.0116*
Irregular	13(59.1)	9(40.9)	
Regular	71(86.6)	11(13.4)	
Postoperative shape			0.7300*
Irregular	71(79.8)	18(20.2)	
Regular	13(86.7)	2(13.3)	
Apoplexy			0.1928
No	43(86.0)	7(14.0)	
Yes	41(75.9)	13(24.1)	

^*^Fisher’s exact test.

**Table 5:** Univariate analysis of categorical variables using chi-square and Fisher’s exact tests: association between factors when postoperative volume difference (O2 − A2) ≥1 SE (1.40 mL) and preoperative volume difference (O1 − A1) <1 SE (1.54 mL).

Characteristics	Postoperative volume difference (O2 − A2) ≥1 SE and preoperative difference (O1 − A1) <1 SE, n (%)		
	No	Yes	P value
Extent of resection			0.0061
Gross total	51(96.2)	2(3.8)	
Subtotal	40(78.4)	11(21.6)	
High grade cavernous sinus invasion			0.3353*
No	29(93.6)	2(6.4)	
Yes	62(84.9)	11(15.1)	
Suprasellar extension			0.6860*
No	15(93.8)	1(6.2)	
Yes	76(86.4)	12(13.6)	
Preoperative shape			0.9999*
Irregular	19(86.4)	3(13.6)	
Regular	72(87.8)	10(12.2)	
Postoperative shape			0.6865*
Irregular	77(86.5)	12(13.5)	
Regular	14(93.3)	1(6.7)	
Apoplexy			0.8821
No	44(88.0)	6(12.0)	
Yes	47(87.0)	7(13.0)	

^*^Fisher’s exact test.

**Table 6:** Univariate analysis of categorical variables using chi-square and Fisher’s exact tests: association between factors when postoperative volume difference (O2 − A2) ≥1 SE (1.40 mL) and preoperative volume difference (O1 − A1) ≥1 SE (1.54 mL).

Characteristics	Postoperative volume difference (O2 − A2) ≥1 SE and preoperative difference (O1 − A1) ≥1 SE, n (%)		
	No	Yes	P value
Extent of resection			0.0054*
Gross total	53(100.0)	0(0.0)	
Subtotal	44(86.3)	7(13.7)	
High grade cavernous sinus invasion			0.1004*
No	31(100.0)	0(0.0)	
Yes	66(90.4)	7(9.6)	
Suprasellar extension			0.5918*
No	16(100.0)	0(0.0)	
Yes	81(92.1)	7(7.9)	
Preoperative shape			<0.0001*
Irregular	16(72.7)	6(27.3)	
Regular	81(98.8)	1(1.2)	
Postoperative shape			0.9999*
Irregular	83(93.3)	6(6.7)	
Regular	14(93.3)	1(6.7)	
Apoplexy			0.1142*
No	49(98.0)	1(2.0)	
Yes	48(88.9)	6(11.1)	

^*^Fisher’s exact test.

**Table 7:** Multivariate logistic regression: association between factors and postoperative volume difference (O2 − A2) ≥1 SE (1.40 mL).

Covariates (model 4.1)	P value	Odds ratio (95% CI)
Preoperative volume difference (O1 − A1), ≥1.54 vs. <1.54 mL	0.3160	2.285(0.454–11.491)
Extent of resection, subtotal vs. gross total resection	0.0071	9.446(1.839–48.510)
High grade cavernous sinus invasion, yes vs. no	0.3777	2.192(0.383–12.526)
Suprasellar extension, yes vs. no	0.9559	1.069(0.102–11.236)
Apoplexy, yes vs. no	0.3089	1.848(0.556–6.036)
Preoperative shape, irregular vs. regular shape	0.2831	2.122(0.537–8.383)
